# Breast Cancer Treatment Decreases Serum Levels of TGF-β1, VEGFR2, and TIMP-2 Compared to Healthy Volunteers: Significance for Therapeutic Outcomes?

**DOI:** 10.3390/pathophysiology29030042

**Published:** 2022-09-01

**Authors:** Varvara Krasnikova, Maria Pospelova, Olga Fionik, Tatyana Alekseeva, Konstantin Samochernykh, Nataliya Ivanova, Nikita Trofimov, Tatyana Vavilova, Elena Vasilieva, Albina Makhanova, Samwel Tonyan, Alexandra Nikolaeva, Evgeniya Kayumova, Maxim Shevtsov

**Affiliations:** 1 Personalized Medicine Centre, Almazov National Medical Research Centre, 2 Akkuratova Str., 197341 Saint Petersburg, Russia; 2 Department of Radiation Oncology, Technishe Universität München (TUM), Klinikum Rechts der Isar, Ismaninger Str. 22, 81675 Munich, Germany; 3 Laboratory of Biomedical Nanotechnologies, Institute of Cytology of the Russian Academy of Sciences (RAS), Tikhoretsky Ave., 4, 194064 Saint Petersburg, Russia; 4 Laboratory of Biomedical Cell Technologies, Far Eastern Federal University, 690091 Vladivostok, Russia

**Keywords:** breast cancer survivors, post-mastectomy pain syndrome, breast cancer, fibrosis molecules, TGF-β1, VEGFR2, TIMP-2, post-radiation fibrosis, breast cancer-related lymphedema

## Abstract

Various complications from a breast cancer treatment, in the pathogenesis of which excessive tissue fibrosis plays a leading role, are a common pathology. In this study, the levels of TGF-β1, VEGFR-2, and TIMP-2 were determined by the immuno-enzyme serum analysis for patients during the long-term period after breast cancer treatment as potential markers of fibrosis. The single-center study enrolled 92 participants, which were divided into two age-matched groups: (1) 67 patients following breast cancer treatment, and (2) 25 healthy female volunteers. The intergroup analysis demonstrated that the patients after breast cancer treatment showed a decrease in the serum levels of TGF-β1 (U = 666, *p* < 0.001) and TIMP-2 (U = 637, *p* < 0.001) as compared to the group of healthy volunteers. The levels of VEGFR-2 in these groups were comparable (U = 1345, *p* = 0.082). It was also found that the type of treatment, the presence of lymphedema, shoulder joint contracture, and changes in lymphoscintigraphy did not affect the levels of TGF-β1, VEGFR-2, and TIMP-2 within the group of patients after breast cancer treatment. These results may indicate that these biomarkers do not play a leading role in the maintenance and progression of fibrosis in the long-term period after breast cancer treatment. The reduced levels of TGF-β1 and TIMP-2 may reflect endothelial dysfunction caused by the antitumor therapy.

## 1. Introduction

Worldwide, breast cancer is the most common type of cancer among women. Due to modern breast cancer screening and the development of novel treatment modalities, the 5-year survival rate in breast cancer reaches 89% [1]. However, more patients develop a number of complications after treatment, which significantly reduce the quality of life and disrupt social functioning [2]. The most common consequences of complex antitumor therapy are secondary lymphedema of the upper limb [3], persistent pain syndrome on the side of the operation [4], polyneuropathy [5], and biomechanical disorders of the upper shoulder girdle [6]. One of the possible pathogenetic mechanisms of developing these complications may be excessive fibrosis of soft tissues caused by surgical treatment and subsequent radiation therapy.

The pathogenetic mechanisms of fibrosis development associated with oncological treatment have not been fully studied. One of the hypotheses is the occurrence of endothelial dysfunction due to the direct and indirect effects of radiation therapy on the vascular wall [7]. A direct effect on the endothelial barrier is realized due to oxidative DNA damage, changes in vascular permeability [8], accelerated aging and apoptosis of cells [9], and induction of the proinflammatory phenotype of the endothelium [10]. Indirect mechanisms of ionizing radiation effect on the endothelium are associated with the activation of mast cells and an increase in vascular permeability [11], as well as with hemolysis that in turn leads to hemoglobin denaturation and subsequent cell damage caused by free iron ions [12]. Furthermore, the apoptotic death and disruption of the functioning of endothelial cells cause damage to the mechanisms of maintaining tissue homeostasis and the deposition of collagen in perivascular spaces, which leads to chronic hypoxia and activation of fibrogenesis [13].

The following hypothesis links the development of fibrous changes with a violation of lymphodynamics due to direct damage to the lymphatic bed during surgery [14]. After the removal of regional lymph nodes, some patients have a systemic progressive violation of the integrity of the lymphatic vessels of the entire limb [15], which leads to the release of protein molecules into the interstitial space. In the later stages, the pathological process becomes irreversible—hyperplasia and obstruction of lymphatic capillaries have been noted [16], which further increases endolymphatic pressure and closes the vicious circle of the pathogenesis of secondary lymphedema [17]. Thus, chronic aseptic inflammation mediated by Th-helpers occurs in subcutaneous adipose tissue [18]. In addition, the active synthesis of proinflammatory interleukins by immune cells leads to a change in the functioning of fibroblasts, increased collagen production, and decreased remodeling of the extracellular matrix [19]. Thus, lymphatic edema may be a separate factor causing the development of soft-tissue fibrosis. The application of less traumatic treatment technologies has led to a decrease in the frequency of lymphatic edema. However, even with the use of a signal lymph node biopsy, secondary lymphedema develops, on average, in 6% of cases [20].

Modern studies point to mesenchymal cells as the primary substrate for the occurrence of fibrosis after exposure to a pathogen [21]. Under ionizing radiation, fibroblasts are activated and transformed into myofibroblasts, which actively secrete various extracellular matrix components. In addition, there is a mechanism for recruiting resting stromal fibroblasts and proliferation of fibroblast progenitor cells [22]. Epithelial–mesenchymal and endothelial–mesenchymal transitions also play an essential role in fibrogenesis—the transformation of damaged endothelial and epithelial cells into fibroblasts, their migration to the affected area, and transition to activated myofibroblasts [23].

The variety of mechanisms of fibrogenesis after oncological treatment makes it difficult to find effective means of therapy and prevention. Moreover, blocking only one of the mechanisms—mesenchymal, endothelial, or inflammatory—will have a limited effect due to the preservation of the alternative pathway. Thus, modern research aims to find a key mediator of fibrogenesis, the inhibition of which will prevent a cascade of pathological reactions leading to excessive extracellular matrix synthesis [22]. In the current study, we assessed serum levels of three biomarkers associated with fibrogenesis: TGF-β1, VEGFR-2, and TIMP-2.

One of the promising potential mediators of fibrosis development is transforming growth factor β1 (TGF-β1), a pleiotropic molecule that regulates proliferation, differentiation, apoptosis, adhesion, and migration of various cells. TGF-β1 is synthesized by platelets, T-lymphocytes, macrophages, endothelial cells, keratinocytes, smooth muscle cells, and fibroblasts [24]. To date, TGF-β1 is considered a key mediator in triggering the pathogenetic mechanisms of fibrosis development [25]. The biological function of TGF-β1 is to activate the proliferation of fibroblasts, the transformation of fibroblasts into myofibroblasts, and the initiation of the epithelial–mesenchymal transition, as well as to increase the synthesis of extracellular matrix and block signals that contribute to its destruction [25]. The TGF-β1 molecule is excreted from cells in an inactive state bound to the latent TGF-β-binding proteins (LTBPs) to form large latent complex (LLC) [26]. Dissociated TGF-β1 from the LLC exerts its activity via the Smad pathways [27].

Studies show that TGF-β1 plays a particular role in implementing all pathways of radiation damage to tissues and is one of the key mediators of pathological fibrogenesis. In particular, it has been proven that reactive oxygen species formed during irradiation can initiate the synthesis of TGF-β1 and contribute to the transition of the latent form of the molecule to the active one due to the destruction of noncovalent bonds between TGF-β1 and LTBPs [28]. Moreover, one of the mechanisms of radiation-mediated aging and apoptosis of endothelial cells is realized through the TGF-β1 signaling pathway [29]. In addition, many inflammatory markers, the level of which increases due to the formation of a proinflammatory phenotype of the endothelium, also contribute to the synthesis of TGF-β1 by fibroblasts [30]. Thus, TGF-β1 is one of the key mediators that trigger a pathological cascade of events, resulting in tissue fibrosis. A number of experimental studies are aimed at developing a specific TGF-β1 blocker for the prevention and treatment of soft-tissue fibrosis caused by various pathogens [31,32]. In a study by Puthawala et al., inhibition of the activation factor TGF-β1 reduced the severity of post-radiation pulmonary fibrosis in mice [33], which was further confirmed by later studies [34]. In addition, a direct relationship was found between lymphedema and TGF-β1 levels in patients with secondary lymphedema of the lower extremities [35]. It should be noted that the marker levels were studied in most studies in the acute damage period [36]. The authors found no scientific papers where the level of TGF-β1 was studied in the long-term period after various treatment regimens for breast cancer. Thus, given the crucial role of the biomarker in the development and progression of fibrosis, TGF-β1 may be a promising molecule for the detection and prevention of late complications of treatment.

Vascular endothelial growth factor receptor 2 (VEGFR-2) is a transmembrane proangiogenic receptor with tyrosine kinase activity that mediates the crucial effects of vascular endothelial growth factor (VEGF) [37]. VEGFR-2 is expressed mainly on vascular endothelial cells, as well as on lymphatic endothelial cells [38]. The primary role of the VEGF–VEGFR system is the formation of new vessels during physiological and pathological processes [39]. In particular, VEGFR-2 is responsible for proliferation, migration, and survival of endothelial cells and vascular permeability during the angiogenesis [40]. The potential role of the VEGF–VEGFR system in complications developing after cancer treatment is being actively studied. Thus, in the work of Mei R. Fu et al., it was found that the level of VEGF initially and eight weeks after breast cancer surgery was increased in a group of patients with severe symptoms of lymphedema compared with groups of patients with moderate and mild symptoms [41]. In addition, genetic variations of VEGF-C, VEGFR-2, and VEGFR-3 associated with the development of breast cancer-related lymphedema were found [42]. Thus, the level of VEGF and VEGFR-2 can be a potential marker of the development of lymphedema in patients after radical breast cancer treatment.

Tissue metalloproteinase inhibitors (TIMPs) are a group of tissue-specific endogenous inhibitors of matrix metalloproteinases whose main role is the degradation of the extracellular matrix [43]. Previously, it was believed that the role of TIMPs was limited to the regulation of extracellular matrix metabolism, but in recent years, other functions of these proteins have been studied. TIMPs have various biological effects, including control of cell proliferation, migration, and invasion, anti-angiogenesis, apoptosis, and synaptic plasticity [44]. In particular, the role of type 2 TIMP (TIMP-2) in stopping cell proliferation and inhibiting angiogenesis by blocking VEGF signaling has been demonstrated in a series of in vitro experiments [45]. In a number of studies, there is a violation of the balance between matrix metalloproteinase-2 (MMP-2) and TIMP-2 in the development of diseases characterized by excessive tissue fibrosis (fibroproliferative alterations in the structure of the palmar aponeurosis, keloid scars) [46,47]. In addition to the effect on vasculogenesis and fibrogenesis, the effect of TIMP-2 expression on the structure and functions of skeletal muscles was found—sarcopenia developed in the experimental model when this molecule was inhibited [48]. Thus, given the leading pathways of fibrogenesis in patients after breast cancer treatment, TIMP-2 may be a promising biomarker of fibrotic changes and a potential target for therapeutic agents.

Taking into consideration the significant role of these molecules (i.e., TGF-β1, VEGF-R2, TIMP-2) in fibrogenesis, we assessed the serum levels of the forementioned molecules in breast cancer patients (n = 67) who developed various complications of BC treatment in the long-term follow-up period (>12 months).

## 2. Materials and Methods

### 2.1. Experimental Design

The study was carried out in compliance with the principles of the Helsinki Declaration of the World Medical Association with the consent of the Ethics Committee of the Federal State Budgetary Institution “Almazov National Medical Research Center” of the Ministry of Health of the Russian Federation (conclusion of 31 October 2019).

#### 2.1.1. Inclusion Criteria

Women aged 25 to 50 after modified unilateral mastectomy or sector mastectomy and radio-chemotherapy who developed post-treatment symptoms associated with cancer-treated breasts but not with primary cancerous lesions were included in the study [49]. Other criteria also included the ECOG performance status of 0–1 and the absence of cardiac, endocrine, rheumatic neuromuscular, or musculoskeletal disorders and other tumors. The age-matched group of healthy female volunteers included women with no history of cancer or severe somatic diseases. All women included in the study signed written informed consent.

#### 2.1.2. Exclusion Criteria

Exclusion criteria included: signs of progression of the main oncological disease; the presence of distant metastases of breast cancer; acute injuries of the musculoskeletal system; the presence of hemodynamically significant atherosclerotic stenosis of the head and neck main arteries; acute infectious and mental diseases, as well as other conditions that prevent examination and manual diagnosis; pregnancy; decompensated somatic pathology; contraindications to lymphoscintigraphy.

**Figure 1:**
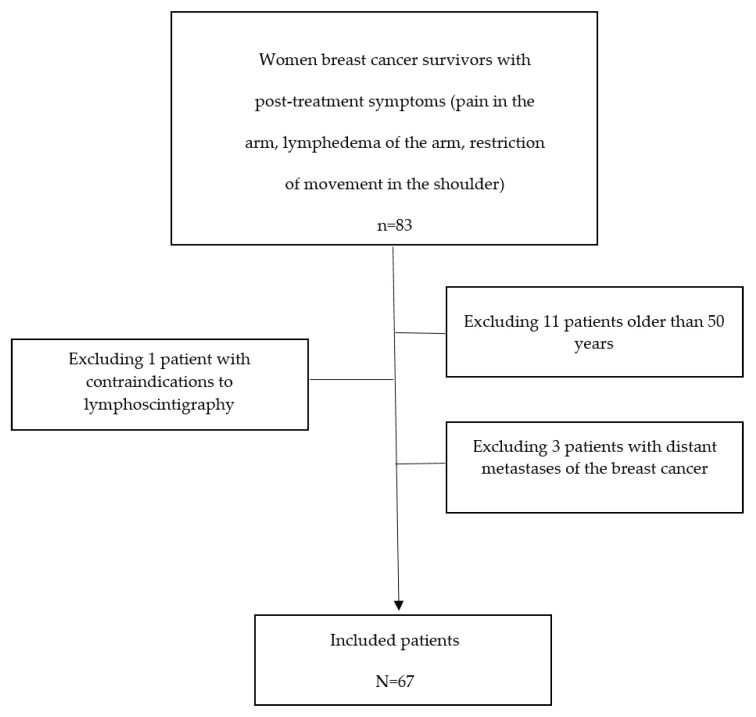
The flowchart of the patient selection process.

The flowchart of the patient selection process is presented in Figure 1.

### 2.2. Clinical Assessment

Clinical assessment included: assessment of complaints; anamnesis; measurement of the volume of the upper extremities; and joint movements. At the initial examination, complaints were collected from patients after breast cancer treatment. The anamnesis included the type, hormone receptor status, major pathological grades, and TNM stage of the breast cancer, the period after the operation, the type of operation, the course of chemotherapy, the course of radiation therapy, the presence of relapses, and the hormonal therapy with Tamoxifen^®^. (Sandoz, Basel, Switzerland).

The assessment of the movements in the shoulder joint on the side of the operation was performed using a goniometer and compared with the movement on the contralateral side.

The upper extremities were measured on both sides to assess the volume of the limbs at seven levels and subsequently to assess the degree of edema. The classification based on determining the difference in the volume of an edematous limb compared to a healthy limb describes four degrees of edema: 0—subclinical condition; I—an increase in the circumference of the affected limb by less than 20%; stage II—an increase of 21–40%; stage III—an increase of more than 40% [50].

Upper extremity lymphedema and/or limitation of movement in the shoulder joint were used as clinical criteria for fibrosis.

### 2.3. Assessment of the Serum Levels of TGF-β1, VEGF-R2, and TIMP-2 Molecules

The serum (of 7 mL blood) was collected from oncological patients’ and healthy volunteers’ blood, aliquoted, and stored at −70 °C. Assessment of soluble transforming growth factor-beta 1 (TGF-β1); soluble receptors 2 for vascular endothelial growth factor (VEGF-R2), and tissue inhibitor of metalloproteinases 2 (TIMP2) was performed using the commercially available Human TGF-β1 ELISA (both Bender MedSystems GmbH, Wien, Austria; Cat. No. BMS249-4), Human VEGF-R2 ELISA (both Bender MedSystems GmbH, Wien, Austria; Cat. No. BMS2019), and Human TIMP-2 Quantikine ELISA (both R&D Systems, Minneapolis, MN, USA; Cat. No. DTM200) according to the manufacturers’ protocols.

### 2.4. Upper Limb Lymphoscintigraphy

Lymphoscintigraphy of the upper extremities was performed using a modified method of manufacturing a radiopharmaceutical agent (RFP), technetium (99mTs) phytate, and subsequent fixation of its passage through the lymphatic bed of the extremities using a scintillation gamma camera in the volume “whole body” to determine the functional state of the lymphatic bed of the upper extremities. In all patients included in the study, a change in the functioning of the lymphatic bed of the upper limb was detected on the side of surgical treatment. In this regard, according to the results of lymphoscintigraphy, the patients were divided into two subgroups: gross changes in the microcirculatory bed (criterion: backflow phenomenon) and compensatory changes (a decrease in the accumulation of RFP in the axillary lymph nodes, the presence of enlarged lymphatic collectors, collaterals, insertion lymph nodes) [51]. The backflow phenomenon was used as an instrumental criterion for fibrosis.

### 2.5. Statistical Analysis

Statistical data were processed using the IBM SPSS Statistics 28.0.1.0 program (IBM, Armonk, New York, NY, USA). All available data were analyzed statistically. To assess the qualitative variables, absolute and relative indicators (% of the number of observations) were used. Quantitative variables were characterized by medians and ranges of values (Me (25 Percentile; 75 Percentile)). Statistical comparison of changes in quantitative indicators to baseline parameters was carried out using nonparametric methods. The statistical significance of changes in quantitative indicators was checked using the Kruskal–Wallis test. The Mann–Whitney U-test was used as a post hoc test. The linear relationship between continuous variables was determined using Spearman’s correlation. *p*-Values under 0.05 were considered statistically significant.

## 3. Results

### 3.1. Clinical Evaluation of Patients

In total, 67 patients following breast cancer therapy and 25 age-matched healthy female volunteers were enrolled in the single-center controlled clinical trial. Patients and healthy women were comparable in age. All women included in the study were Caucasian. All patients were in the late postoperative period (>12 months) after radical treatment of breast cancer (Table 1).

All patients had clinical manifestations of treatment complications (Table 2).

When analyzing the results of lymphoscintigraphy, two subgroups were additionally identified: patients with severe changes in the lymphatic bed without clinical manifestations of lymphedema and patients without pronounced changes in the lymphatic bed with clinical lymphedema. The results of lymphoscintigraphy are presented in Table 3.

### 3.2. TGF-β1, VEGFR-2, and TIMP-2 Serum Levels

**Figure 2:**
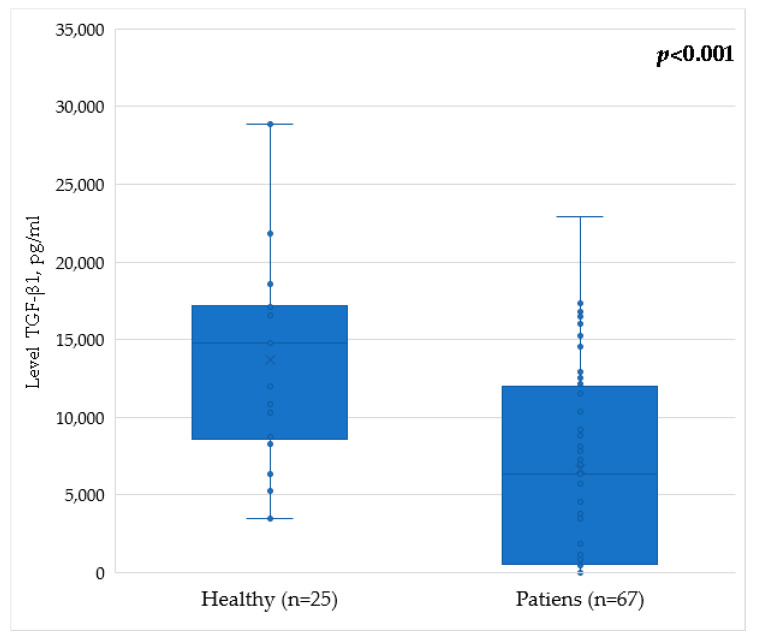
Level of TGF-β1 in the serum of patients after breast cancer treatment and of healthy volunteers.

TGF-β1 serum levels in healthy donors constituted 17,374 [8802; 17,152] pg/mL. In the group of patients following breast cancer treatment, the serum levels of TGF-β1 were 6356 [551; 11,706] pg/mL. In an intergroup comparison, patients after breast cancer treatment showed a statistically significant decrease in the level of TGF-β1 molecules (Table 4). For clarity, the results are also demonstrated in Figure 2.

**Figure 3:**
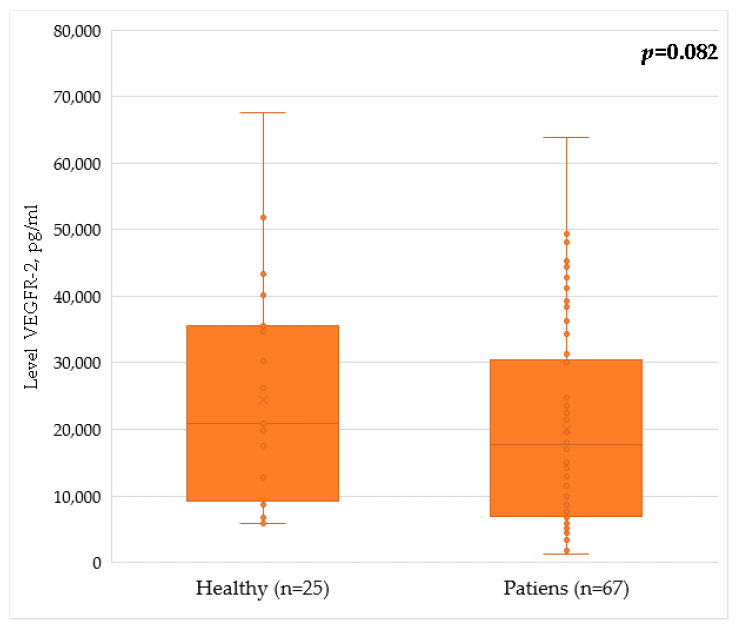
Serum level of VEGFR-2 in patients after breast cancer treatment and in healthy volunteers.

VEGFR-2 serum levels in healthy donors were 20,850 [10,137; 35,402] pg/mL. In the group of patients following breast cancer treatment, the serum levels of VEGFR-2 were 17,750 [6865; 30,200] pg/mL. Intergroup comparison in patients after breast cancer treatment did not reveal statistically significant differences in the level of VEGFR-2 molecules compared with healthy women (Table 4). For clarity, the results are also demonstrated in Figure 3.

**Figure 4:**
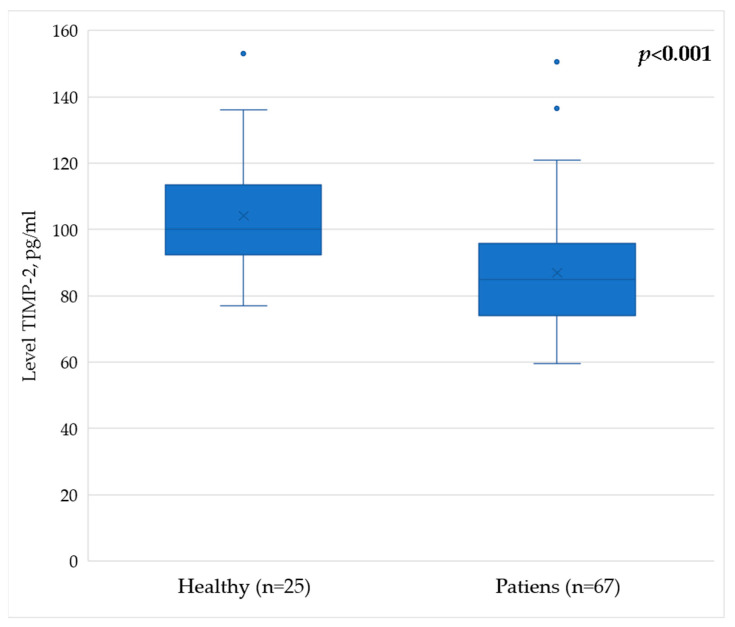
Serum level of TIMP-2 in patients after breast cancer treatment and in healthy volunteers.

TIMP-2 serum levels in healthy donors constituted 100 [92; 113] pg/mL. In the group of patients following breast cancer treatment, the serum levels of TIMP-2 were 85 [74; 95] pg/mL. In an intergroup comparison, patients after breast cancer treatment showed a statistically significant decrease in the level of TIMP-2 molecules (Table 4). For clarity, the results are also demonstrated in Figure 4.

Patients after breast cancer treatment were divided into subgroups according to the following characteristics: the presence of lymphedema, limitation of movement in the shoulder joint, hormone receptor status of breast cancer, major pathological grades of breast cancer, operation type, and history of radiation therapy and chemotherapy. All subgroups of patients were compared between themselves and healthy volunteers using the Kruskal–Wallis test. The level of fibrosis molecules and statistical results of the analysis are presented in Table 5.

As can be seen from Table 5, statistically significant differences in the levels of TGF-β1 and TIMP-2 were obtained for all studied characteristics when comparing subgroups of patients with healthy volunteers. The level of VEGFR2 did not have statistically significant differences in any of the characteristics.

The results of the post hoc analysis levels of TGF-β1 and TIMP-2 are presented in Table 6 and Table 7, respectively.

As can be seen from Table 6 and Table 7, patients with and without lymphedema had statistically significantly lower levels of TGF-β1 and TIMP-2 than healthy volunteers. No significant intergroup differences were found.

Moreover, patients with and without limitation of movement in the shoulder joint had a statistically significantly lower level of TGF-β1 and TIMP-2 than healthy volunteers. However, no significant intergroup differences were found.

Patients with hormone receptor-positive status of breast cancer had a lower level of TGF-β1 than healthy volunteers. At the same time, in patients with hormone-negative breast cancer, there were no statistically significant differences in the level of TGF-β1 when compared with HP+ patients and healthy volunteers.

Both hormone-positive and hormone-negative breast cancer patients had lower TIMP-2 levels than healthy volunteers, with no intergroup differences found among patients.

As can be seen from Table 6 and Table 7, patients whose regimen of chemotherapy was included had lower levels of TGF-β1 and TIMP-2 than healthy volunteers. At the same time, in patients with only surgery and a combination of surgery and radiation therapy, the levels of TGF-β1 and TIMP-2 were comparable to the control group. Moreover, no significant intergroup differences were found in patients depending on the type of treatment.

Patients with modified unilateral mastectomy Madden and sector mastectomy had statistically significantly lower levels of TGF-β1 and TIMP-2 than healthy volunteers. No significant intergroup differences were found.

Moreover, patients with various changes in lymphodynamics had a lower level of TGF-β1 and TIMP-2 than healthy volunteers, while no significant intergroup differences were found.

### 3.3. Correlation Analysis of the Level of Fibrosis Molecules

Correlation analysis of the dependence of the level of fibrosis molecules on the age of patients, the period after treatment, and among themselves was performed. A statistically significant inverse correlation was found between the period after treatment and the level of TIMP-2 (ρ = −0.317, *p* = 0.010). An inverse correlation was also found between the level of TIMP-2 and the level of VEGFR-2 (ρ = −0.369, *p* = 0.002). A direct correlation was also found between the level of TIMP-2 and the level of TGF-β1 (ρ = 0.328, *p* = 0.008), and an inverse correlation was found between the level of VEGFR2 and the level of TGF-β1 (ρ = −0.264, *p* = 0.034). There was no significant correlation between the period after surgery and the levels of VEGFR-2 (ρ = 0.105, *p* = 0.403) and TGF-β1 (ρ = 0.09, *p* = 0.474) or between the level of serum biomarkers and the age of patients (TGF-β1: ρ = 0.093, *p* = 0.463; VEGFR-2: ρ = −0.055, *p* = 0.662; TIMP-2: ρ = −0.13, *p* = 0.919).

The results of the correlation analysis are presented in Table 8.

## 4. Discussion

Our study found statistically significantly lower serum levels of TGF-β1 and TIMP-2 in patients in the long-term follow-up period (>12 months) after breast cancer treatment than in healthy female volunteers, while the level of VEGFR-2 was comparable in these groups. TGF-β1 [52], TIMP-2 [53], and VEGFR-2 [54] have been recognized in a number of studies as the key regulators of fibrogenesis and angiogenesis, which determined the choice of these biomarkers for detecting the effects of breast cancer treatment.

All the patients included in the study had various local complications of antitumor treatment, including the swelling of the upper limb on the side of the operation, restriction of movements in the shoulder joint, and objectively detected changes in lymphodynamics. Considering the fundamental role of fibrosis in the development of these complications [55], the authors suggested that, in patients with pronounced fibrotic changes, the levels of TGF-β1 and TIMP-2 will be higher than in healthy volunteers, and the level of VEGFR-2, which is a powerful proangiogenic factor [56], will be lower. However, the results of the current study completely refute these assumptions, which allows us to modify the ideas about the pathogenesis of complications of breast cancer treatment.

The decrease in the level of TGF-β1 and TIMP-2 in the study group may be caused by several factors. First of all, at the moment, the long-term mechanisms of maintaining fibrosis after oncological treatment have not been studied in detail. In the acute period of damage, TGF-β1 and TIMP-2 play a leading role, initiating the mechanisms of fibroblast activation and extracellular matrix synthesis [36]. However, the role of these molecules in the long-term period after treatment is not so obvious. Presumably, the progression of fibrogenesis occurs due to the involvement of alternative molecular pathways: activation of fibroblasts due to increased levels of proinflammatory molecules [57], impaired regulation of microRNA [58], and endothelial dysfunction [59]. In this case, the reduced level of fibrosis regulators may be associated with the activation of sanogenetic processes and the suppression of available pathways of fibrogenesis to reduce pathological tissue remodeling.

It is also necessary to consider the various biological effects of TGF-β1 and TIMP-2. For example, in addition to fibrogenetic and proinflammatory activity, TGF-β1 may have an angioprotective function [60]. Indeed, TGF-β1 inhibits the proliferation of vascular smooth muscle cells and thus reduces the remodeling of the vascular network [61], stabilizes the vascular wall, and prevents its infiltration by lymphocytes [62]. It has also been proven that TGF-β1 suppresses the synthesis of proinflammatory cytokines by the endothelium [63]. Earlier, the authors found a marked increase in intercellular adhesion molecules in the blood serum of patients after breast cancer treatment [64], which confirms the hypothesis of antagonistic interaction of these biomolecules. TIMP-2, being a synergist of TGF-β1, may be included in this interaction scheme. TIMP-2 also plays a role in cognitive processes, synaptogenesis, and aging [65]. The proven decrease in cognitive functions in patients after cancer treatment [66], as well as the acceleration of the trajectory of cell aging [67], may be correlated with a lower level of TIMP-2 in these patients. The same variety of functions can explain the absence of significant intragroup differences in the level of molecules depending on the clinical picture in the main group. It should be assumed that the level of markers is influenced not only by the severity of fibrosis but also by other factors.

It should also be noted that endothelial cells are one of the sources of TGF-β1 [68] and TIMP-2 [69] synthesis. Endothelial dysfunction is characteristic of patients after oncological treatment, including in the long-term period after it [70]. Thus, a decrease in the levels of TGF-β1 and TIMP-2 may be caused by a malfunction of endothelial cells. This hypothesis is confirmed by the fact that, in patients whose treatment regimen included chemotherapy, the level of molecules was lower than in healthy volunteers, while in other patients, it did not significantly differ. Chemotherapy has the most toxic effect on the endothelium [71], which explains the reduced level of markers synthesized by endothelial cells in the blood serum.

Of note is the decrease in the level of TGF-β1 in patients with a history of hormone-positive cancer. Some studies have found a decrease in the level of TGF-β1 in hormone-positive cancer before treatment [72]. Thus, the level of this molecule can also be influenced by the hormonal status of the tumor.

The study found a direct correlation between the levels of TIMP-2 and TGF-β1 and a negative correlation between the levels of TIMP-2 and VEGFR-2. This fact can be explained by the proven synergistic functioning of TIMP-2 and TGF-β1 [73] and antagonistic functioning between TIMP-2 and VEGFR-2 [74] in fibrogenesis and angiogenesis, while VEGFR-2 and TGF-β1 can mediate the synthesis of each other [75]. Moreover, no significant correlations were found between the levels of molecules and the age of patients, while in healthy people, this correlation is present [76]. It is possible that the levels of molecules, in this case, are primarily influenced by other factors, or the small age range of patients included in the study did not allow clear statistical dependencies to form.

The limitations of the study are related to the small sample size and the heterogeneity of the group of patients by type of treatment, as well as the lack of control of the marker level before the start of treatment. In addition, the authors used only indirect signs of fibrosis (i.e., lymphedema, contracture of the shoulder joint, changes in lymphoscintigraphy).

## 5. Conclusions

Thus, it can be assumed that the TIMP-2 and TGF-β1 pathways are not crucial in maintaining and progressing fibrosis in patients after radical breast cancer treatment in the long-term follow-up period of >12 months. This result reveals the need for further study of this topic to find effective ways to correct complications of antitumor therapy, including in the long-term period after it. It is likely that the decrease in the level of these markers after antitumor therapy reflects endothelial dysfunction that persists in the long-term period after treatment.

## Figures and Tables

**Table 1:** Characteristics of the patients.

Group Characteristics of Patients	Patients after Breast Cancer Treatment n = 67	Healthy n = 25
Age (years)	47.0 [44; 49]	42.0 [38; 47]
Years since treatment	3.0 [2; 5]	-
Number of patients TNM stage		
I (T1N0M0)	8 (12%)	-
II A (T2N1M0)	46 (68%)	-
II B (T3N1M0)	3 (5%)	-
III A (T3N2M0)	2 (3%)	-
III B (T4N2M0)	8 (12%)	-
Types of breast cancer		
Ductal carcinoma in situ (DCIS)	7 (11%)	-
Invasive ductal carcinoma (IDC)	49 (73%)	-
Invasive lobular carcinoma (ILC)	11 (16%)	-
Breast cancer hormone receptor status		
Hormone receptor-positive (HR+)	55 (72%)	-
Hormone receptor-negative (HR−)	12 (18%)	-
Major pathological grades of breast cancer		
Grade 1	10 (15%)	-
Grade 2	35 (52%)	-
Grade 3	22 (33%)	-
Treatment of breast cancer		
Complex treatment (surgical treatment, radiotherapy, chemotherapy)	37 (55%)	-
Combination of surgical treatment and chemotherapy	18 (27%)	-
Combination of surgical treatment and radiotherapy	7 (10%)	-
Only surgical treatment	5 (7%)	-
Type of surgical treatment		
Modified unilateral mastectomy Madden	53 (79%)	-
Sector mastectomy	14 (21%)	-
Hormonal therapy (tamoxifen vs. GH-LH analogues)		
Do not take the medicine	12 (18%)	
Take the medicine	50 (75%)	
Completed the course	5 (7%)	

**Table 2:** Clinical characteristics in breast cancer survivors.

Clinical Characteristics	Number of Patients (N, %)
Restriction of movement in the shoulder	37 (55%)
Lymphedema of the arm	27 (40%)

**Table 3:** Results of lymphoscintigraphy in breast cancer survivors.

Change Type	Number of Patients (N, %)
Dermal backflow	36 (53%)
Compensatory changes	31 (47%)
Dermal backflow without clinical lymphedema	10 (15%)
Clinical lymphedema without dermal backflow	8 (12%)

**Table 4:** Fibrosis molecules in the serum of patients following breast cancer treatment and healthy volunteers, pg/mL.

Fibrosis Molecules	Patients (n = 67)	Healthy (n = 25)	Mann–Whitney U-Test	Significance (*p*)
TGF-β1	6356 [551; 11,706]	17,374 [8802; 17,152]	666	<0.001 *
VEGFR2	17,750 [6865; 30,200]	20,850 [10,137; 35,402]	1345	0.082
TIMP-2	85 [74; 95]	100 [92; 113]	637	<0.001 *

*—differences between the groups were significant at *p* < 0.05.

**Table 5:** Serum biomarker levels in the study subgroups, pg/mL.

Sign of Separation	Characteristic of the Sign	Number of Patients (and Age)	TGF-β1	Kruskal–Wallis Test	*p*	VEGFR2	Kruskal–Wallis Test	*p*	TIMP-2	Kruskal–Wallis Test	*p*
Presence of lymphedema (LE)	yes	27 (42.0 [40; 46])	6087 [1065; 1008]	32.231	<0.001 *	16,328 [8224; 21,223]	3.127	0.209	82 [75; 92]	30.749	<0.001 *
	no	40 (48.0 [46; 50])	6498 [292; 11,229]			19,050 [6086; 31,110]			88 [72; 99]		
Limitation of movement in the shoulder joint (LSh)	yes	37 (47.5 [43.5; 49])	8277 [3502; 9223]	22.589	<0.001 *	9223 [7559; 12,122]	2.328	0.312	91 [77; 101]	22.589	<0.001 *
	no	30 (44.6 [41; 46.5])	6932 [1823; 8993]			16,468 [8014; 25,183]			81 [74; 97]		
Hormone receptor status of breast cancer (HRS)	HR+	55 (42.0 [39; 47.4])	6356 [512; 10,133]	21.62	<0.001 *	18,250 [6749; 28,800]	4.344	0.227	86 [75; 96]	24.397	<0.001 *
	HR−	12 (47.4 [44; 49])	6230 [1950; 1284]			13,355 [9022; 29,200]			81 [71; 101]		
Major pathological grades of breast cancer (G)	G1	10 (48.0 [43; 49])	6041 [627; 8749]	34.061	<0.001 *	22,750 [12,248; 36,509]	4.274	0.233	73 [69; 82]	43.015	<0.001 *
	G2	35 (47.0 [44; 48])	6340 [870; 9628]			17,450 [6321; 24,950]			91 [82; 101]		
	G3	22 (46.0 [42; 49])	4811 [864; 12,505]			15,132 [10,096; 20,100]			81 [72; 87]		
Treatment history	Only surgical treatment (OS)	5 (45.0 [43; 48.7])	7941 [7437; 14,676]	37.287	<0.001 *	6086 [4373; 23,050]	2.261	0.262	93 [85; 102]	36.643	<0.001 *
	Surgical treatment and radiotherapy (S + R)	7 (46.5 [44; 48])	8868 [7604; 11,705]			3574 [3508; 13,612]			91 [84; 101]		
	Surgical treatment and chemotherapy (S + Ch)	18 (46.0 [43.8; 49])	5725 [959; 8307]			14,391 [6787; 30,200]			81 [68; 94]		
	Complex treatment (CT)	37 (47.0 [44; 49)]	18,250 [9022; 31,995]			18,250 [9022; 31,995]			87 [73; 96]		
Operation type	Modified unilateral mastectomy Madden (M)	53 (45.3 [42.5; 47])	6940 [870; 12,568]	36.297	<0.001 *	18,050 [7255; 27,550]	4.221	0.239	87 [75; 98]	31.876	<0.001 *
	Sector mastectomy (SM)	14 (47,8 [44.8; 49)]	7437 [5460; 11,251]			21,450 [9207; 27,150]			82 [72; 95]		
Lymphoscintigraphy changes	Dermal backflow (DB)	36 (48.0 [45; 49])	4876 [704; 9787]	22.526	<0.001 *	19,650 [8392; 31,529]	4.029	0.258	82 [73; 94]	22.757	<0.001 *
	Compensatory changes (CCh)	31 (46.3 [43.3; 47])	6624 [704; 12,381]			15,058 [5930; 23,350]			83 [74; 101]		
Changes in lymphoscintigraphy with clinic	Dermal backflow without clinical lymphedema (DB without LY)	10 (44.0 [42; 46])	2412 [152; 6349]	21.152	<0.001 *	28,880 [7657; 42,449]	3.567	0.312	89 [75; 95]	23.784	<0.001 *
	Clinical lymphedema without dermal backflow (LY without DB)	8 (47.0 [44; 49)]	6024 [777; 9707]			15,132 [9121; 19,145]			85 [78; 91]		

*—differences between the groups were significant at *p* < 0.05.

**Table 6:** Games–Howell test result for two groups according to TGF-β1 level.

Sign of Separation	(I) Criterion	(J) Criterion	Mean Difference (I-J)	Std. Error	*p*	95% Confidence Interval (CI)	
						Lower Bound	Upper Bound
LY	Healthy	Yes	7076	1528	<0.001 *	3405	10,075
	Healthy	No	6979	1530	<0.001 *	3309	10,650
	Yes	No	−96	1487	1	−3669	3576
LSh	Healthy	Yes	7705	1668	<0.001 *	3670	9293
	Healthy	No	6668	1445	<0.001 *	3205	9223
	Yes	No	−288	4102	0.78	−4799	2727
HRS	Healthy	HR+	7315	1379	<0.001 *	4077	10,553
	Healthy	HR−	5455	2600	0.13	−1392	12,303
	HR+	HR−	−1860	2472	0.74	−8540	4819
G	Healthy	G1	8013	1923	0.005 *	2380	9223
	Healthy	G2	7368	1235	<0.001 *	4128	9223
	Healthy	G3	6836	1647	0.001 *	2394	9223
	G1	G2	−644,	1976	0.98	−6363	5073
	G1	G3	−1177	2257	0.95	−7465	5111
	G2	G3	−532	1709	0.98	−5123	4058
Treatment history	Healthy	OS	2083	3160	0.96	−10,928	15,095
	Healthy	S + R	1648	2576	0.96	−13,882	17,128
	Healthy	S + Ch	5597	1567	0.01 *	1053	10,141
	Healthy	CT	3828	1474	0.04 *	−517	7973
	OS	S + R	−435	3883	1	−15,068	14,196
	OS	S + Ch	3513	3300	0.82	−9210	16,237
	OS	CT	1745	3227	0.98	−11,034	1
	S + R	S + Ch	3949.23	2747.39	0.65	−9768.73	17,667.19
	S + R	CT	2180.70	2695.26	0.91	−11,926.71	16,288.11
	S + Ch	CT	−1768.53	1756.00	0.85	−6802.28	3265.23
Operation type	Healthy	SM	6464.78	1293.53	<0.001 *	3069.14	9860.43
	Healthy	M	5664.95	1825.40	0.03 *	417.75	10,912.15
	M	Healthy	−799.83	1920.63	0.98	−6218.37	4618.70
Lymphoscintigraphy changes	Healthy	DB	7485.27	1548.83	<0.001 *	3397.93	11,572.61
	Healthy	CCh	6453.91	1587.79	<0.001 *	2253.70	10,654.11
	DB	Cch	1031.36	1566.33	0.91	−3117.97	5180.69
Changes in lymphoscintigraphy with clinic	Healthy	DB without LY	9164.26	2095.13	<0.001 *	3198.09	15,130.44
	Healthy	LY without DB	7742.97	2136.88	0.02 *	1455.36	14,030.58
	DB without LY	LY without DB	−1421.30	2547.40	0.94	−8728.40	5885.80

*—differences between the groups were significant at *p* < 0.05.

**Table 7:** Games–Howell test result for two groups according to TIMP-2 level.

Sign of Separation	(I) Criterion	(J) Criterion	Mean Difference (I-J)	Std. Error	*p*	95% Confidence Interval (CI)	
						Lower Bound	Upper Bound
LY	Healthy	Yes	17.52	4.64	<0.001 *	6.38	28.67
	Healthy	No	17.01	4.13	<0.001 *	7.11	26.92
	Yes	No	−0.51	4.48	0.99	−11.30	10.28
LSh	Healthy	Yes	16.58	5.61	0.02 *	2.89	30.27
	Healthy	No	17.61	3.81	<0.001 *	8.46	26.75
	Yes	No	1.03	5.25	0.98	−11.90	13.96
HRS	Healthy	HR+	16.70	3.87	<0.001 *	7.44	25.96
	Healthy	HR−	20.24	6.48	0.02 *	3.35	37.13
	HR+	HR−	3.54	6.20	0.84	−12.94	20.03
G	Healthy	G1	31.24	3.98	<0.001 *	20.22	42.27
	Healthy	G2	13.67	3.86	0.004 *	3.53	23.80
	Healthy	G3	23.29	4.29	<0.001 *	11.84	34.74
	G1	G2	−17.58	4.28	0.002 *	−29.29	−5.86
	G1	G3	−7.95	4.67	0.341	−20.72	4.81
	G2	G3	9.62	4.56	0.165	−2.53	21.78
Treatment history	Healthy	OS	10.97	5.80	0.41	−11.10	33.04
	Healthy	S + R	9.40	10.13	0.87	−59.65	78.45
	Healthy	S + Ch	20.57	4.60	<0.001 *	7.12	34.02
	Healthy	CT	12.07	4.43	0.04 *	−0.43	24.56
	OS	S + R	−1.57	11.18	1.00	−58.42	55.29
	OS	S + Ch	9.60	6.60	0.61	−12.63	31.83
	OS	CT	1.10	6.48	1.00	−20.86	23.06
	S + R	S + Ch	11.17	10.61	0.82	−50.13	72.46
	S + R	CT	2.67	10.54	1.00	−59.57	64.90
	S + Ch	CT	−8.50	5.44	0.53	−24.06	7.06
Operation type	Healthy	SM	17.03	3.99	<0.001 *	6.55	27.51
	Healthy	M	22.73	5.00	<0.001*	8.56	36.90
	M	Healthy	5.70	5.34	0.71	−9.14	20.54
Lymphoscintigraphy changes	Healthy	DB	18.03	4.65	<0.001 *	5.75	30.31
	Healthy	CCh	17.32	4.36	<0.001 *	5.77	28.87
	DB	Cch	0.71	4.72	1.00	−11.78	13.20
Changes in lymphoscintigraphy with clinic	Healthy	DB without LY	17.14	5.97	0.04 *	0.03	34.25
	Healthy	LY without DB	17.50	5.45	0.03 *	1.67	33.33
	DB without LY	LY without DB	0.36	6.85	1.00	−19.24	19.97

*—differences between the groups were significant at *p* < 0.05.

**Table 8:** Correlation analysis of the levels of fibrosis molecules.

	TGF-β1	VEGFR2	TIMP-2	Age (Years)	Years since Treatment
TGF-β1	-	ρ = −0.264, *p* = 0.034 *	ρ = 0.328, *p* = 0.008 *	ρ = 0.093, *p* = 0.463	ρ = 0.09, *p* = 0.474
VEGFR2	ρ = −0.264, *p* = 0.034 *	-	ρ = −0.369, *p* = 0.002 *	ρ = −0.055, *p* = 0.662	ρ = 0.105, *p* = 0.403
TIMP-2	ρ = 0.328, *p* = 0.008 *	ρ = −0.369, *p* = 0.002 *	-	ρ = −0.13, *p* = 0.919	ρ = −0.317, *p* = 0.010 *

*—differences between the groups were significant at *p* < 0.05.

## Data Availability

Not applicable.
